# The Hospitals Association

**Published:** 1891-10-31

**Authors:** 


### The Hospitals Association.

The Ninth Session of the Hospitals Association opened on Wednesday, the 21st inst., at Charing Cross Hospital, when the first general meeting was held under the presidency of Mr. H. Cozens-Hardy, Q.C., M.P., who took the chair at 8 p.m. Among those present who listened to a most interesting and exhaustive paper by Mr. L. S. Bristowe, M.A., Barrister-at-Law, on "The Mortmain and Charitable Uses Act, 1891," were Dr. J. S. Bristowe (President), Mr. Arthur Hulme (Queen's Hospital, Birmingham), Mr. W. Irving Cook, Mr. G. A. Cross, Mr. A. D. Tyssen, D.C.L., Mr. Mark Whitwill (Bristol Children's Hospital), Mr. A. W. Moore, Mr. Edwin Howard (the Master of St. Katherine, Regent's Park), Mr. P. Michelli (Hon. Secretary), Mr. J. C. Steele (Chairman of Executive Committee), Sir William Moore, K.C.I.E., Surgeon-Major Ince, M.D., Mr. Thomas Ryan (St. Mary's Hospital), &c.

In the discussion which followed, Mr. A. D. Tyssen, in criticising the new Act, contended that, admitting the necessity for a change in the law—a point upon which he was not at all convinced himself—the limit of time in which land devised to a charity had to be sold should be longer than a year, and should date from the time the charity actually became possessed of the propert y. Referring to clauses 7 and 8, he thought that some sim pler method might have been devised, in the case where land was left for the actual site or occupation of charity, than having to move the High Court for sanction to retain such land; and he also considered that some direction should have been contained in the Act, whereby the responsibility of registering a bequest of land with the Charity Commissioners should attach to those having the execution of the will. No such provision had been made, and in his opinion it was most desirable that the Charity Commissioners should have official intimation of such bequests.

Mr. Thomas Ryan (St. Mary's Hospital) referring to the highly satisfactory circumstances in which they found themselves, celebrating as they were the completion of this admirable and useful work for the benefit of charities throughout the country, desired to bring home to those directing hospitals and kindred institutions the value of co-operation as exemplified in the successful passing of the new Act. He urged hospital managers and all interested in hospital matters to support the Hospitals Association heartily, and by thus combining enable the Council to do yet further good work for our charities.

The Chairman, in summing up and thanking Mr. Bristowe for his paper, expressed the pleasure it had given him to have been associated with the work of carrying this most desirable reform. Combating Mr. Tyssen's criticisms, Mr. Cozens-Hardy pointed out the difficulty there would have been in getting the Bill through Parliament with too many details, and on the whole he thought the new Act was altogether the best measure that they could have hoped for, bearing in mind the exigencies of Parliament. Referring to Mr. Tyssen's remarks as to the absence of any direction to apprise the Charity Commissioners of land bequeathed to a charity, he (Mr. Cozens-Hardy) would just say this, that as a matter of fact, the Charity Commissioners themselves had actually drawn the clauses referring to themselves, and the Act as it stood was approved of by the Commissioners.

A vote of thanks to the Chairman closed the proceedings.

